# Complications of calcaneo-stop procedure in pediatric flexible flatfoot: a systematic review and meta-analysis

**DOI:** 10.1097/BPB.0000000000001355

**Published:** 2026-05-22

**Authors:** Vito Pavone, Gianluca Testa, Marco Sapienza, Marco Simone Vaccalluzzo, Maria Gaia Dugo, Francesco Leonforte, Alessia Caldaci, Federico Canavese, Maurizio De Pellegrin

**Affiliations:** a Department of General Surgery and Medical Surgical Specialties, Section of Orthopaedics, A.O.U. Policlinico Rodolico-San Marco, University of Catania; b Department of Integrated Hygiene, Organizational, and Service Activities (Structural Department), Health Management, University Hospital Polyclinic ‘G. Rodolico – San Marco’, Catania; c DISC-Dipartimento di Scienze Chirurgiche e Diagnostiche Integrate, University of Genova; d Department of Orthopedic and Traumatology, IRCCS Istituto Giannina Gaslini, Genova; e San Raffaele Telethon Institute for Gene Therapy (SR-TIGET), San Raffaele Scientific Institute, Milan, Italy

**Keywords:** calcaneo-stop, complications, flexible flatfoot, meta-analysis, pediatric foot surgery, subtalar arthroereisis

## Abstract

The calcaneo-stop procedure is a minimally invasive surgical technique widely used to treat symptomatic flexible flatfoot (FFF) in pediatric patients. Although generally considered safe, complication rates vary across studies, and no quantitative synthesis has specifically evaluated its adverse event profile. A systematic review and meta-analysis were conducted according to Preferred Reporting Items for Systematic Reviews and Meta-Analyses 2020 guidelines. Five databases were searched up to May 2025. Studies reporting postoperative complications after calcaneo-stop in patients younger than 18 years were included. Pooled complication rates were calculated using random-effects models, with subgroup analyses based on age, sex, screw type, and diameter. Twenty-two studies involving 1777 patients and 3082 operated feet were included. The overall pooled complication rate was 7.97% (95% confidence interval: 5.96–9.97%). The most frequent complications were postoperative pain (2.79%), reintervention (2.27%), incision-site symptoms (2.23%), peroneal spasm (1.72%), screw breakage (1.42%), superficial infection (1.37%), and screw loosening (0.72%). Higher complication rates were observed in patients older than 13 years (15.84%; *P* < 0.001), in cortical screws (13.2%), and in screws greater than or equal to 4.5 mm in diameter (13.9%; *P* = 0.0019). Bioabsorbable screws and 6.5 mm implants showed the lowest complication rates. The calcaneo-stop procedure appears to be a safe and effective treatment for symptomatic pediatric FFF, with complication risk influenced by patient age and implant characteristics.

## Introduction

Flexible flatfoot (FFF), or pes planus, is a common orthopedic condition in children and adolescents, characterized by a partial or complete collapse of the medial longitudinal arch, hindfoot valgus, and forefoot abduction during weight-bearing. While often considered physiological during early childhood, persistent or symptomatic cases may lead to pain, fatigue, altered gait, reduced participation in physical activities, and long-term postural imbalances [1–3].

The stability of the medial arch is ensured by the interaction of bony structures, ligaments, and dynamic components such as the posterior tibial tendon. The plantar fascia, spring ligament, and deltoid ligament provide passive support, whereas the posterior tibial tendon acts as the primary dynamic stabilizer during gait [4,5]. Alterations in these structures can compromise foot biomechanics, resulting in excessive subtalar pronation and progressive flattening of the arch [6].

The prevalence of flatfoot in pediatric populations is estimated between 10 and 25%, with higher rates observed in younger children and declining with age because of musculoskeletal maturation [3,7–9]. Factors such as ligamentous laxity, increased BMI, neuromuscular conditions, male sex, and improper footwear have been associated with persistent flatfoot or symptomatic presentations [3,7,10].

In most children, FFF resolves spontaneously and does not require treatment. However, in cases where symptoms persist beyond 8–10 years of age, or when functional impairment occurs, surgical correction may be considered [11,12]. Various surgical options are available, including calcaneal osteotomies (e.g. Evans, medializing displacement calcaneal osteotomy), tendon transfers (e.g. flexor digitorum longus transfer), or arthrodesis procedures. These techniques, though effective, are often more invasive and involve longer recovery times or limitations in joint mobility [13–15].

Among minimally invasive approaches, the calcaneo-stop procedure has gained popularity for the treatment of symptomatic FFF in skeletally immature patients. This technique, classified as a subtalar extra-articular arthroereisis, involves the percutaneous insertion of a screw into the sinus tarsi to mechanically block excessive eversion of the subtalar joint, restoring physiological alignment and supporting the medial arch [2,12,16]. It is frequently performed bilaterally, does not require bone cutting, and is associated with faster recovery and lower morbidity compared with traditional osteotomies [12,16,17].

Despite its advantages, the calcaneo-stop procedure is not free from complications. Reported adverse events include postoperative pain, implant loosening or breakage, peroneal muscle spasm, superficial infections, scar-related symptoms, and, in rare cases, the need for revision surgery [16–20]. Reported complication rates are highly heterogeneous across the literature, and definitions vary widely, complicating the assessment of its true safety profile.

Previous systematic reviews have primarily focused on functional outcomes or radiographic improvements [2,12,21], while no high-level evidence has provided a focused quantitative analysis of postoperative complications in this population. A thorough understanding of the type, frequency, and predictors of complications is essential for guiding clinical decision-making, optimizing implant selection, and improving patient counseling.

The present systematic review and meta-analysis aims to evaluate the prevalence and characteristics of postoperative complications after the calcaneo-stop procedure in children and adolescents. Furthermore, subgroup analyses will explore potential associations with patient age, sex, screw type, and screw diameter, thereby providing clinically relevant data to support evidence-based surgical planning.

## Material and methods

This systematic review and meta-analysis was conducted in accordance with the Preferred Reporting Items for Systematic Reviews and Meta-Analyses (PRISMA 2020) guidelines.

### Search strategy

A comprehensive literature search was performed across five electronic databases: PubMed, Embase, Web of Science, ScienceDirect, and the Cochrane Library. The search was conducted up to May 2025, using a combination of keywords and medical subject headings terms: (‘calcaneostop’ OR ‘subtalar arthroereisis’) AND (‘flatfoot’ OR ‘pes planus’) AND (‘children’ OR ‘pediatric’) AND (‘complications’). Boolean operators (AND/OR) were used to optimize sensitivity, and no language or publication-year restrictions were applied. Duplicates were removed using EndNote and automated tools before screening.

### Eligibility criteria

Studies were included if they met the following criteria:

1. Population: pediatric patients (<18 years) with symptomatic FFF.
2. Intervention: surgical correction with the calcaneo-stop procedure (subtalar extra-articular arthroereisis).
3. Outcomes: reporting of postoperative complications (overall or specific).
4. Study design: prospective or retrospective cohort studies.

Exclusion criteria were:

1. Case reports, narrative reviews, technical notes, or expert opinion.
2. Studies involving adult patients or procedures other thancCalcaneo-stop.
3. Lack of extractable complication data.

### Study selection and data extraction

**Fig. 1:**
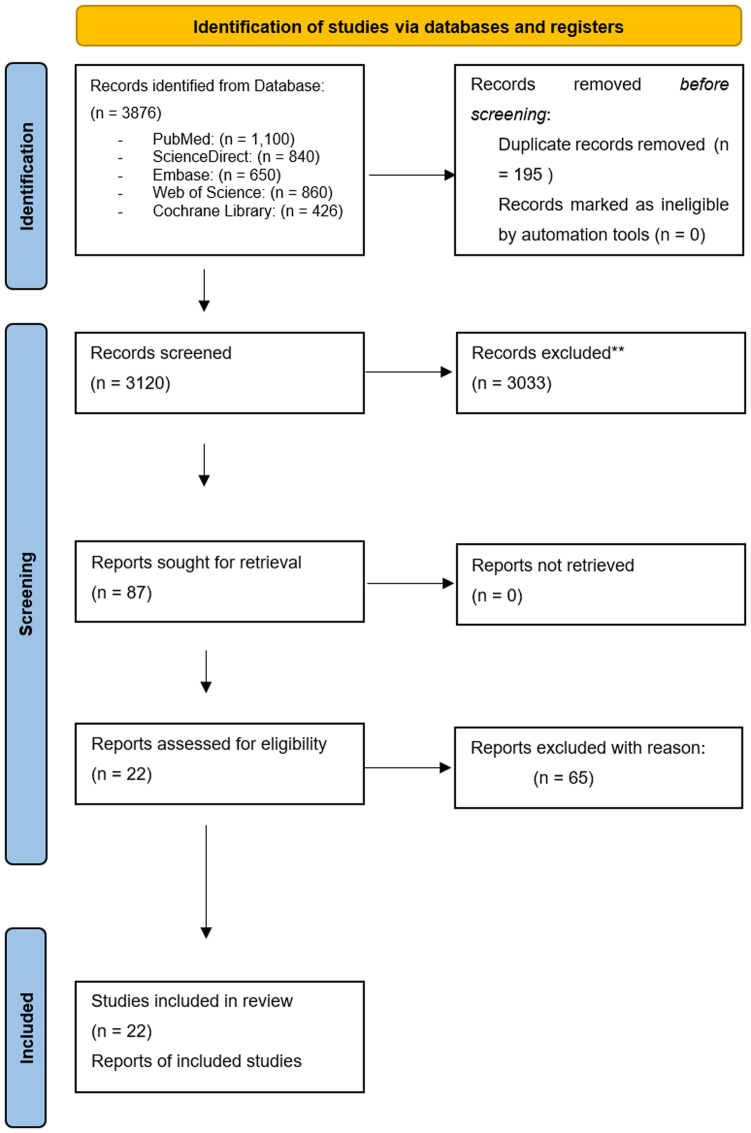
PRISMA 2020 flow diagram. Diagram summarizing the study selection process, including identification, screening, eligibility assessment, and final inclusion of studies in the systematic review and meta-analysis. PRISMA, Preferred Reporting Items for Systematic Reviews and Meta-Analyses.

Titles and abstracts were screened independently by two reviewers (M.S.V. and M.G.D.), followed by full-text assessment for eligibility. Discrepancies were resolved through discussion or consultation with a third reviewer. The study selection process was summarized using a PRISMA 2020 flow diagram, which details the number of records identified, screened, excluded, and included in the final analysis (Fig. 1).

For each included study, the following data were extracted:

1. General information: author, year, country, study design.
2. Patient demographics: number of patients and feet, age, sex, follow-up duration.
3. Surgical variables: screw type and diameter, use of bilateral procedures, implant removal.
4. Reported complications: overall and by type (e.g. pain, screw loosening, breakage, infection, peroneal spasm, reintervention).

If complication data were missing or not explicitly reported, a conservative assumption of zero events was applied, as per protocol. This approach minimizes the risk of overestimating event rates but may underestimate complication frequency, which is acknowledged as a limitation.

### Statistical analysis

Meta-analyses of proportions were performed using random-effects models for each complication type to account for interstudy variability. Pooled prevalence and 95% CIs were calculated. Heterogeneity was assessed using the *I*^2^ statistic, with values greater than 50% indicating substantial heterogeneity.

Subgroup analyses were conducted to investigate the impact of:

1. Age group (<11, 11–13, >13 years).
2. Sex (male vs. female).
3. Screw type (cancellous, cortical, bioabsorbable, others).
4. Screw diameter (≤4.5, 6.5, 7–9 mm).

Statistical comparisons between subgroups were performed using the *χ*^2^ test, with a significance level set at *P* less than 0.05.

All analyses were conducted using RevMan 5.4, Python (SciPy, Matplotlib, Seaborn), and Microsoft Excel for consistency checks and visualizations (forest plots, bar charts).

## Results

### Eligibility criteria

A total of 22 studies published between 1996 and 2025 were included in this systematic review and meta-analysis. These studies encompassed 1777 pediatric patients undergoing the calcaneo-stop procedure for symptomatic FFF, accounting for a total of 3082 treated feet. The average age at surgery was 11.2 years, with reported age ranges spanning from 4 to 17 years across studies. The mean follow-up period was 62.7 months (range: 12–187 months), with most studies reporting outcomes over at least 2 years.

Sex distribution was reported in the majority of studies, though a few lacked detailed data. The male-to-female ratio varied but tended to be relatively balanced overall. Most studies focused on children aged 8–14, reflecting the typical age range for surgical correction of FFF.

A comprehensive summary of the study characteristics, including sample size, number of treated feet, age distribution, sex ratio, and follow-up duration, is presented in Table 1.

**Table 1:** Demographic characteristics of included studies

Authors	Patients (*n*)	Feet (*n*)	Mean age (range)	Sex (M : F)	Follow-up (months)
Silva *et al*. [22]	336 (39)	644	11.7 ± 1.3	139 : 197	41.3
Almeida *et al*. [23]	26 (30)	44	11.5 (6–15)	15 : 11	30.2
Ghaznavi *et al.* [24]	44 (40)	57	10.23	27 : 17	12.0
Vogt *et al.* [25]	25 (41)	36	12 (8–16)	15 : 10	27.0
Zahid *et al.* [26]	15 (42)	30	8.87 (5–14)	10 : 5	114.0
Elmarghany *et al.* [27]	42 (43)	84	9.92 (7–15)	26 : 16	29.1
Kubo *et al.* [28]	50 (44)	95	11.3 (5–15)	NR	35.8
Memeo *et al.* [29]	101 (45)	202	13.6 (8–16)	NR	130.0
Pavone *et al.* [2]	68 (2)	136	12.7 (9–15)	38 : 30	57.6
Caravaggi *et al.* [30]	13 (46)	13	11.3	NR	12.5
Giannini *et al.* [31]	44 (33)	88	11.7 (8–14)	31 : 13	56.0
Arbab *et al.* [32]	41 (47)	73	11.8 (9–14)	23 : 18	30.6
Das *et al.* [33]	15 (48)	25	12.5	10 : 5	54.0
Samaila *et al.* [34]	36 (37)	54	11.8 (8–16)	22 : 14	89.0
Calvo Calvo *et al.* [35]	52 (49)	103	11.6 (7–14)	32 : 20	187.0
Abubeih *et al.* [36]	26 (34)	45	12.08 (7–14)	9 : 17	35.6
De Pellegrin *et al.* [37]	485 (50)	732	11.5 (5–17)	267 : 218	54.0
Richter and Zech [38]	18 (51)	31	10.6 (8–12)	8 : 10	30.0
Pavone *et al.* [39]	242 (29)	410	11 (7–14)	157 : 85	88.0
Koning *et al.* [40]	40 (52)	80	8 (4–11)	22 : 5	151.0
Jerosch *et al.* [41]]	18 (53)	21	11.9 (8–14)	13 : 5	31.0
Prieto Álvarez *et al.* [42]	40 (54)	79	10 (5–17)	17 : 23	12.0
Total/mean	1777	3082	≈11.2 years	–	62.7

### Postoperative complications: general overview

**Fig. 2:**
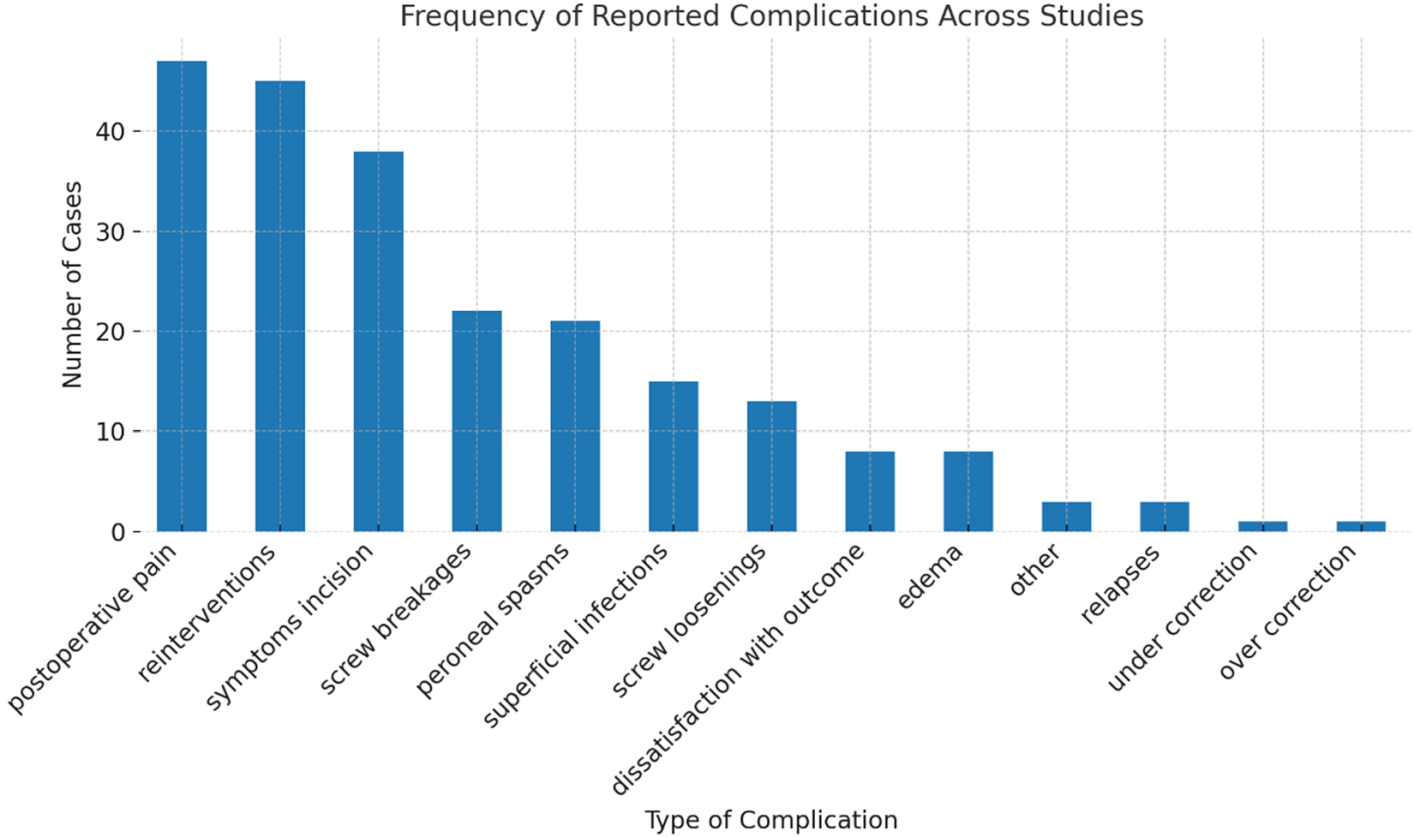
Frequency of complications associated with the calcaneo-stop procedure. Bar chart displaying the number of complications reported across 22 studies. The most common complications included postoperative pain (*n* = 47), unplanned reinterventions (*n* = 45), and symptoms at the incision site (*n* = 38).

Across the 22 included studies, a total of 227 postoperative complications were reported, corresponding to a crude overall complication rate of 7.37% (227 complications over 3082 feet). The relative frequencies of each complication are summarized in Fig. 2, which visually highlights that postoperative pain and implant-related events constitute the majority of adverse outcomes.

Definitions of complications, including postoperative pain and surgical site–related symptoms, were recorded as described in the original studies. Because of heterogeneity in reporting, no uniform time threshold could be applied to distinguish early postoperative discomfort from late complications. Events were therefore analyzed according to the definitions provided by each study’s authors.

Meta-analyses of proportions were performed for the most frequently reported adverse events. The pooled prevalence rates and 95% CIs were as follows:

1. Postoperative pain: 2.79% (95% CI: 1.90–3.67).
2. Unplanned reinterventions: 2.27% (95% CI: 0.70–3.84).
3. Incision site symptoms: 2.23% (95% CI: 1.49–2.98).
4. Screw breakage: 1.42% (95% CI: 0.84–2.01).
5. Peroneal muscle spasm: 1.72% (95% CI: 0.86–2.58).
6. Superficial infection: 1.37% (95% CI: 0.58–2.16).
7. Screw loosening: 0.72% (95% CI: 0.12–1.32).

**Fig. 3:**
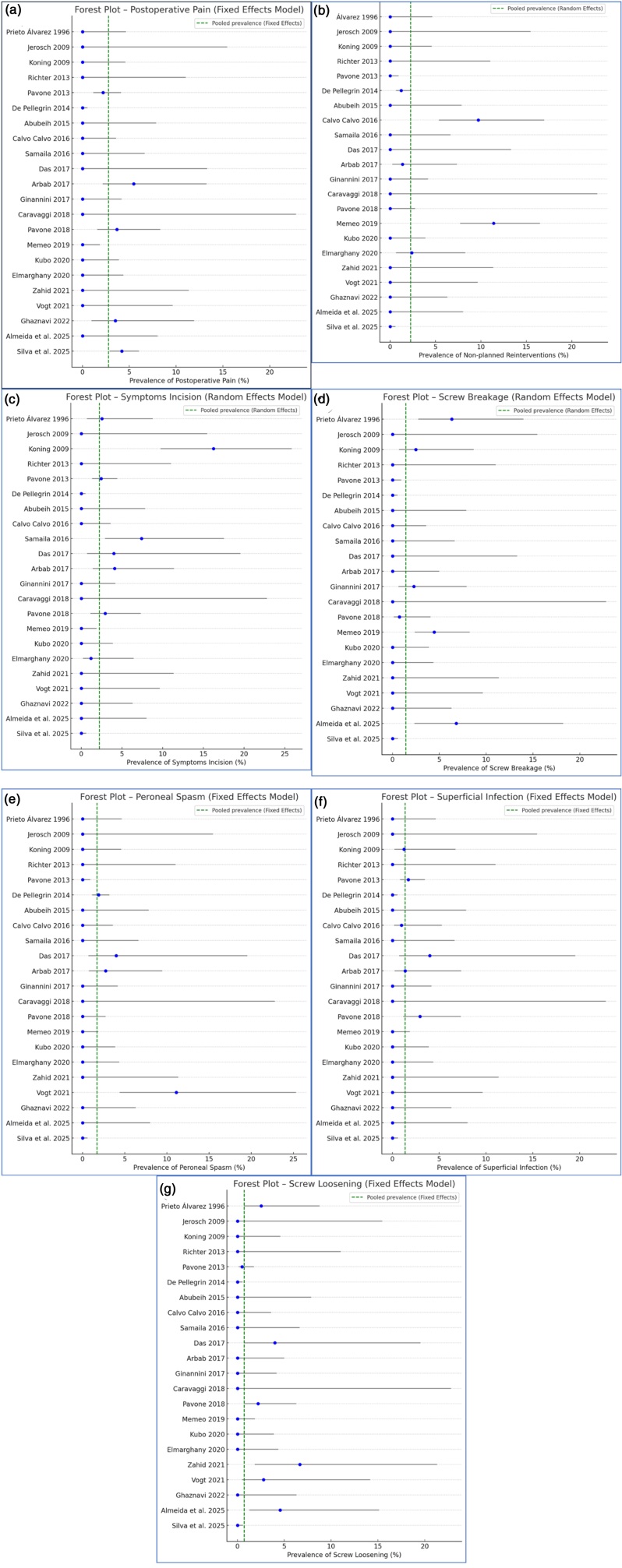
Forest plots – pooled prevalence of postoperative complications (part 1). (a) Postoperative pain. (b) Unplanned reinterventions. (c) Symptoms at the incision site. (d) Screw breakage. Forest plots – pooled prevalence of postoperative complications (part 2). (e) Peroneal muscle spasm. (f) Superficial infection. (g) Screw loosening.

The distribution of complications by study and by type is detailed in Table 2 and Fig. 3a,b.

**Table 2:** Summary of postoperative complications

Authors	Patients (*n*)	Feet (*n*)	Complications (*n*)	Type of complications
Silva *et al*. [22]	336	644	35	27 Postoperative pain, eight dissatisfaction with outcome
Almeida *et al*. [23]	26	44	6	Two screw loosenings, three screw breakages, one under correction
Ghaznavi *et al.* [24]	44	57	2	Two postoperative pain
Vogt *et al.* [25]	25	36	5	One screw loosening, four peroneal spasms
Zahid *et al.* [26]	15	30	2	Two screw loosenings
Elmarghany *et al.* [27]	42	84	3	One symptoms incision, two reinterventions
Kubo *et al.* [28]	50	95	0	No complications
Memeo *et al.* [29]	101	202	32	Nine screw breakages, 23 reinterventions
Pavone *et al.* [2]	68	136	17	Three screw loosenings, one screw breakages, five postoperative pain, four symptoms incision, four superficial infections
Caravaggi *et al.* [30]	13	13	0	No complications
Giannini *et al.* [31]	44	88	2	Two screw breakages
Arbab *et al.* [32]	41	73	12	Four postoperative pain, two peroneal spasms, three symptoms incision, one over correction, one reintervention, one superficial infection
Das *et al.* [33]	15	25	4	One screw loosening, one peroneal spasm, one symptoms incision, one superficial infection
Samaila *et al.* [34]	36	54	4	Four symptoms incision
Calvo Calvo *et al.* [35]	52	103	11	10 reinterventions, one superficial infection
Abubeih *et al.* [36]	26	45	0	No complications
De Pellegrin *et al.* [37]	485	732	34	14 Peroneal spasms, nine reinterventions, eight edema, three other
Pavone *et al.* [39]	242	410	31	Two screw loosenings, nine postoperative pain, 10 symptoms incision, three relapses, seven superficial infections
Richter and Zech [38]	18	31	0	No complications
Koning *et al.* [40]	40	80	16	Two screw breakages, 13 symptoms incision, one superficial infection
Jerosch *et al.* [41]]	18	21	0	No complications
Prieto Álvarez *et al.* [42]	40	79	11	Two screw loosenings, five screw breakages, two symptoms incision
Total	1777	3082	227	

### Meta-analysis of overall complication rate

A meta-analysis of proportions was performed to estimate the pooled prevalence of complications. Using a random-effects model to account for clinical and methodological heterogeneity among studies, the overall complication rate was estimated at 7.97% (95% CI: 5.96–9.97%).

**Fig. 4:**
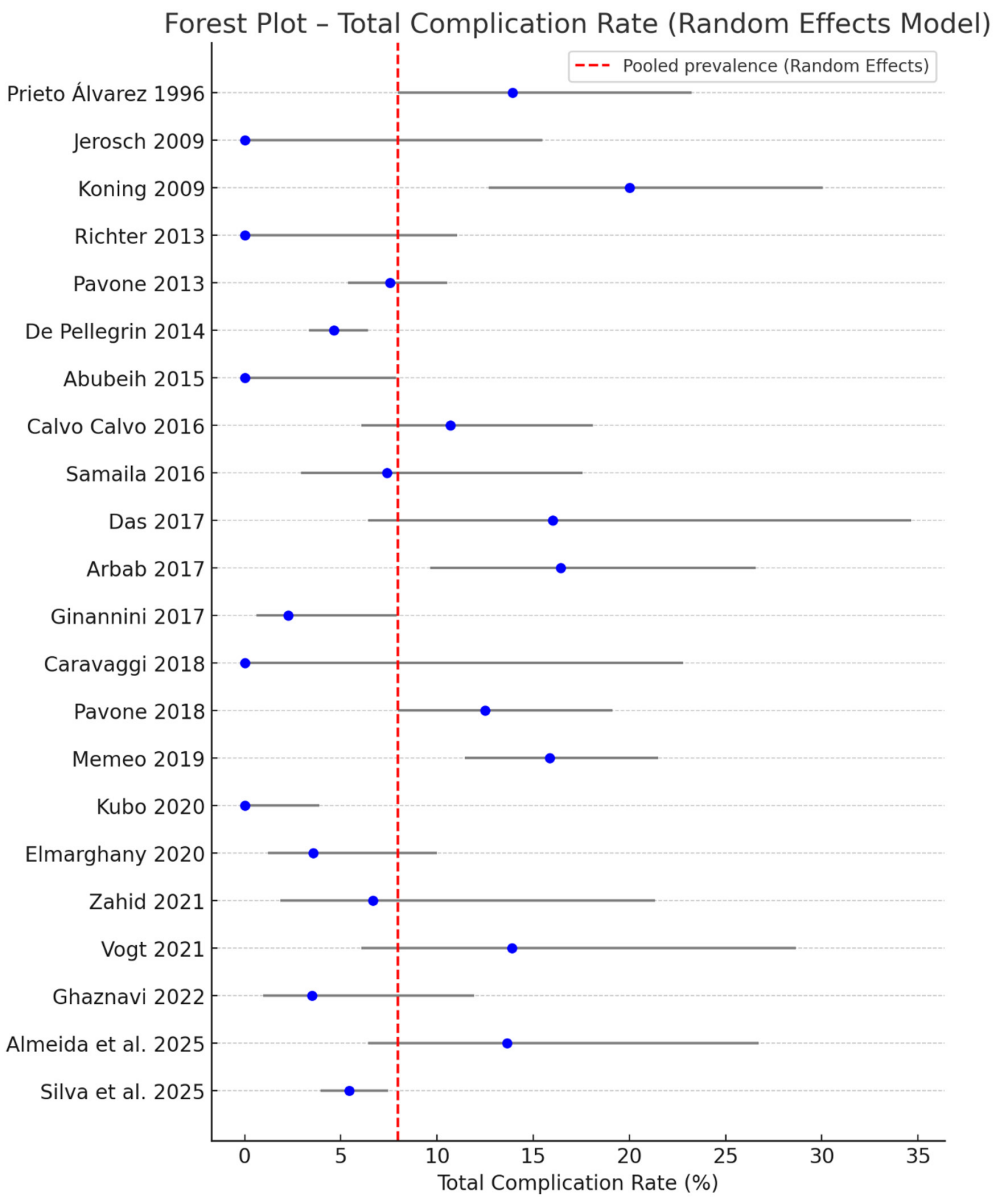
Forest plot – overall complication rate. Meta-analysis of proportions showing the pooled complication rate with individual study estimates and 95% CIs. CI, confidence interval.

The results are presented in Fig. 4 as a forest plot showing individual study estimates with corresponding CIs, as well as the pooled estimate. Although heterogeneity was moderate, all included studies contributed to the estimation with no outliers or extreme values identified.

### Subgroup analyses

#### Age-based differences

Studies were grouped based on the reported mean age of the population at the time of surgery into three categories:

1. Group A: less than 11 years (49 complications/663 feet, 7.39%).
2. Group B: 11–13 years (124 complications/1673 feet, 7.41%).
3. Group C: greater than 13 years (32 complications/202 feet, 15.84%)

Age subgroups were defined according to the distribution most commonly reported among included studies, with 11 years used as a threshold to distinguish younger patients and 13 years for complication analysis. These cutoffs approximately correspond to the preadolescent and early adolescent stages, within the generally accepted ideal age range (9–14 years) for the calcaneo-stop procedure in symptomatic FFF.

**Fig. 5:**
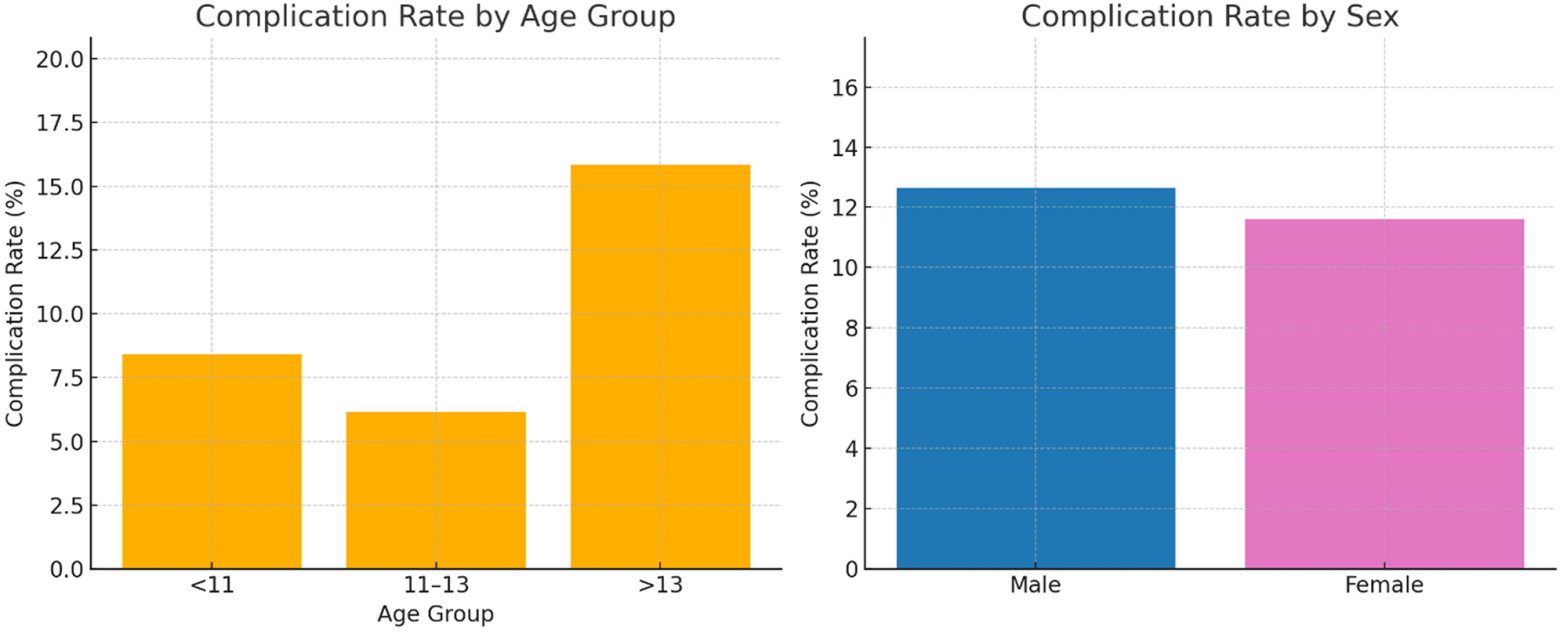
Complication rates by age and sex. Left panel: bar chart showing increased complication rate in patients older than 13 years. Right panel: similar complication rates in male and female patients, with no statistically significant difference.

The incidence of complications was significantly higher in group C compared with groups A and B (*P* < 0.001), suggesting that older age at the time of surgery may be associated with increased risk. This could reflect reduced skeletal plasticity or more advanced deformity at presentation. The comparative complication rates across age groups are visualized in Fig. 5.

#### Sex-based differences

Among the 19 studies that reported sex-specific data, a total of 881 male and 719 female patients were analyzed. Complication rates were estimated by proportional distribution of cases, resulting in:

1. Male patients: 111.4 complications (12.65%).
2. Female patients: 83.6 complications (11.62%).

The difference between groups was not statistically significant (*P* = 0.586), suggesting that sex is not a major predictor of postoperative complications following the calcaneo-stop procedure.

### Influence of screw type

To evaluate the potential impact of implant type on complication rates, studies were categorized according to the type of screw used: cancellous, cortical, bioabsorbable, or other/unspecified. The corresponding complication rates were:

1. Cancellous screws: 5.8% (96 complications/1649 feet).
2. Cortical screws: 13.2% (66 complications/501 feet).
3. Bioabsorbable screws: 4.7% (four complications/101 feet).
4. Other implants: 14.6% (27 complications/185 feet).

**Fig. 6:**
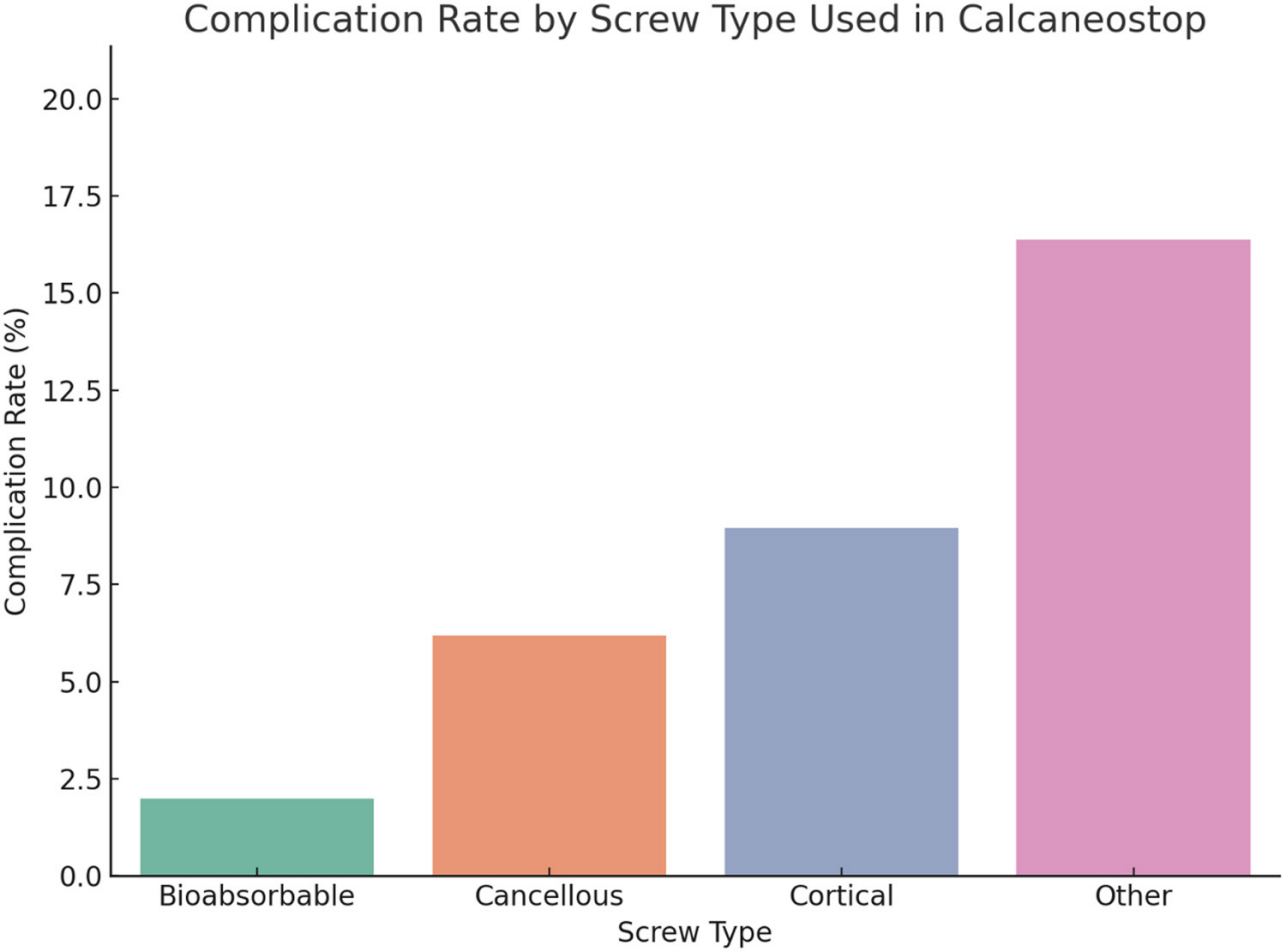
Complication rates by screw type. Bar chart illustrating complication rates stratified by screw type (cancellous, cortical, bioabsorbable, other). Cortical screws were associated with the highest complication rate.

The difference was statistically significant (*P* < 0.001), with cancellous and bioabsorbable screws demonstrating the most favorable safety profiles. These findings are summarized in Table 3 and graphically represented in Fig. 6.

**Table 3:** Distribution of complications by screw type and diameter

Authors	Screw type	Screw diameter	Feet treated	Complications reported
Silva *et al*. [22]	Cancellous	6.5 mm	644	35
Almeida *et al*. [23]	Cancellous	6.5 mm	44	6
Ghaznavi *et al.* [24]	Cancellous	6.5 mm	57	2
Vogt *et al.* [25]	Cancellous	6.5 mm	36	5
Zahid *et al.* [26]	Other	≤4.5 mm	30	2
Elmarghany *et al.* [27]	Cancellous	6.5 mm	84	3
Kubo *et al.* [28]	Cancellous	7–9 mm	95	0
Memeo *et al.* [29]	Cancellous	6.5 mm	202	32
Pavone *et al.* [2]	Cortical	≤4.5 mm	136	17
Caravaggi *et al.* [30]	Bioabsorbable	7–9 mm	13	0
Giannini *et al.* [31]	Bioabsorbable	7–9 mm	88	2
Das *et al.* [33]	Cortical	6.5 mm	25	4
Samaila *et al.* [34]	Cortical	NR	54	4
Abubeih *et al.* [36]	Cancellous	6.5 mm	45	0
De Pellegrin *et al.* [37]	Cancellous	6.5 mm	732	34
Richter and Zech [38]	Cancellous	≤4.5 mm	31	0
Pavone *et al.* [39]	Cortical	≤4.5 mm	410	31
Jerosch *et al.* [41]]	Cancellous	6.5 mm	21	0
Prieto Álvarez *et al.* [42]	Cancellous	6.5 mm	79	11
Arbab *et al.* [32]	NR	NR	73	12
Calvo Calvo *et al.* [35]	NR	NR	103	11
Koning *et al.* [40]	Other	Other	80	16

### Influence of screw diameter

Implants were also stratified based on reported diameter into three categories:

1. Small (≤4.5 mm): 13.9% complication rate (56 complications/402 feet).
2. Medium (6.5 mm): 6.4% (79 complications/1234 feet).
3. Large (7–9 mm): 7.5% (25 complications/332 feet).

**Fig. 7:**
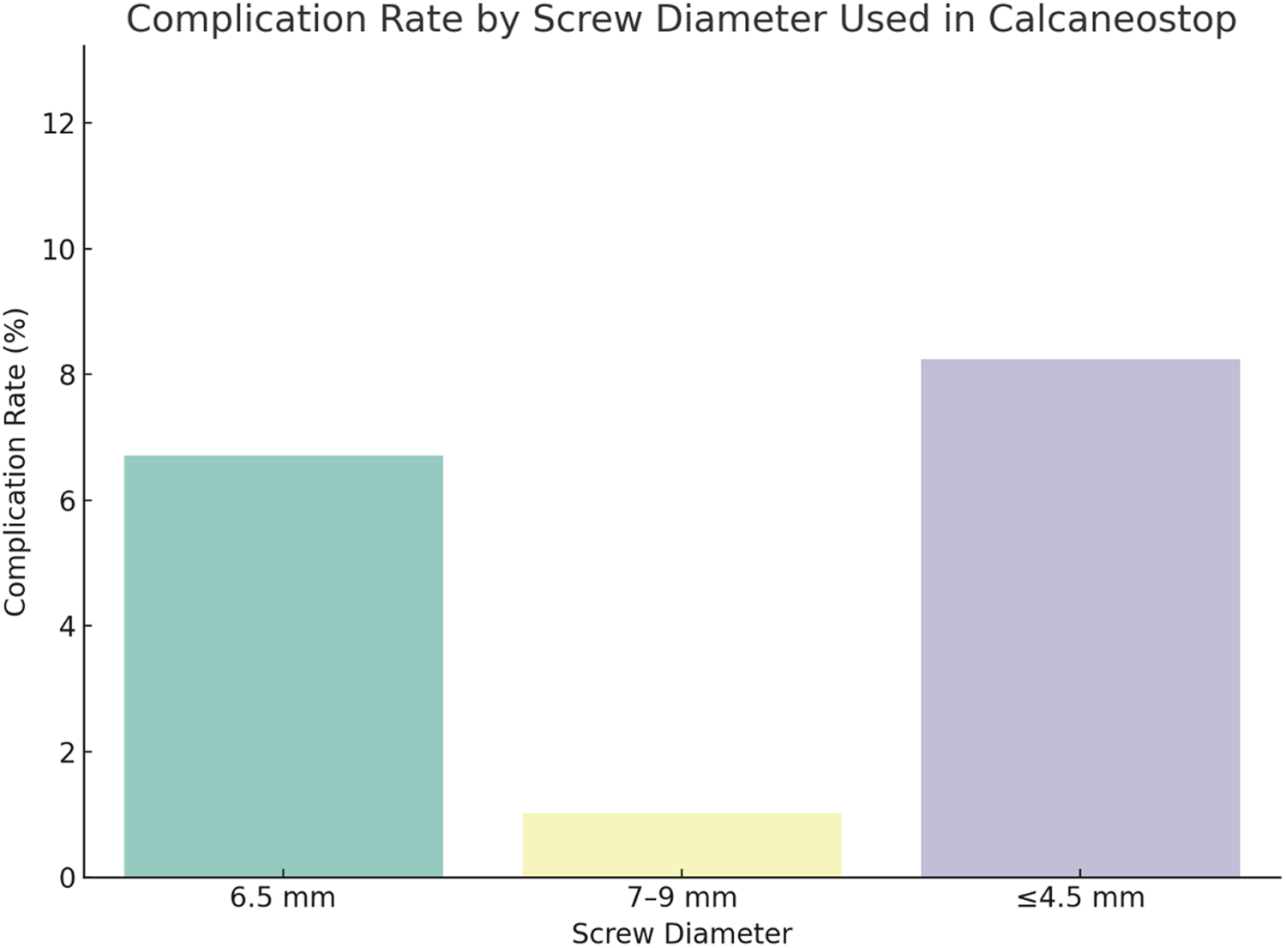
Complication rates by screw diameter. Bar chart showing complication rates for different screw diameters. Screws less than or equal to 4.5 mm showed the highest rate (13.9%), while 6.5 mm screws had the lowest (6.4%).

The smallest screws were associated with a significantly higher risk of complications compared with 6.5 mm and larger screws (*P* = 0.0019), suggesting that undersized implants may lead to insufficient mechanical stability or increased risk of loosening or breakage. These results are illustrated in Fig. 7.

## Discussion

### Summary of main findings

This systematic review and meta-analysis evaluated the complication profile of the calcaneo-stop procedure for the surgical treatment of symptomatic FFF in pediatric patients. Based on data from 22 studies encompassing 3082 operated feet, the overall pooled complication rate was 7.97% (95% CI: 5.96–9.97%). The most frequent adverse events were postoperative pain (2.79%), unplanned reinterventions (2.27%), symptoms at the incision site (2.23%), screw breakage (1.42%), peroneal muscle spasms (1.72%), superficial infections (1.37%), and screw loosening (0.72%).

These findings support the overall safety and efficacy of the calcaneo-stop procedure, particularly in young patients with flexible deformities unresponsive to conservative measures.

### Interpretation and clinical relevance

Postoperative pain emerged as the most common complication. While frequently mild and self-limiting, it may reflect local irritation from the implant, excessive correction, or poor biomechanical adaptation. In some studies, such as those by Silva *et al*. [22] and Pavone *et al*. [2], elective hardware removal effectively resolved symptoms. These findings suggest that routine follow-up and patient education about possible discomfort are essential parts of postoperative care.

Screw-related complications, such as loosening and breakage, although rare, raise concern regarding implant stability. Our subgroup analysis showed that smaller-diameter screws (≤4.5 mm) were significantly associated with a higher rate of complications (13.9%) compared with 6.5 mm screws (6.4%). This finding aligns with Giannini *et al*. [31], who emphasized the role of implant geometry and cortical engagement in achieving optimal fixation. Similarly, cortical screws had a complication rate of 13.2%, compared with 5.8% for cancellous screws and only 4.7% for bioabsorbable devices. These data reinforce the need for careful implant selection based on patient size, bone quality, and skeletal maturity.

Symptoms at the surgical incision, although not typically severe, were reported in over 2% of cases and may impact patient satisfaction, especially in bilateral cases. This emphasizes the importance of atraumatic dissection, precise implant positioning, and proper wound closure in minimizing local soft-tissue irritation.

Peroneal spasms, likely resulting from altered subtalar mechanics or excessive correction, were observed in a small percentage of patients (1.72%). Although most cases resolved with physiotherapy, persistent symptoms may require additional interventions, including muscle relaxants or, in rare cases, surgical release or botulinum toxin injection [34,43].

Superficial infections were rare (1.37%) and managed conservatively. No study reported deep infections or implant-associated osteomyelitis, confirming the low infective risk of this technique, especially when standard sterile precautions and postoperative wound care protocols are applied.

### Subgroup insights: age, sex, and implant variables

Patient age was found to be a significant factor in complication risk. Adolescents older than 13 years exhibited a complication rate of 15.84%, more than double that observed in younger patients. This likely reflects reduced bone plasticity, greater biomechanical demands, and delayed intervention. This is in line with the findings of De Pellegrin *et al*. [37], who reported suboptimal remodeling capacity in older children.

In contrast, sex was not significantly associated with the overall complication rate (*P* = 0.586), a finding consistent with previous literature suggesting similar biomechanical outcomes and tolerance to arthroereisis in both sexes [43,44].

The type and size of the screw significantly influenced outcomes. Larger screws (6.5 mm and above) and bioabsorbable materials yielded better results. These findings have direct implications for implant selection in daily surgical practice, especially in smaller or skeletally immature feet.

### Comparison with existing literature

Our findings corroborate those of Galán-Olleros *et al*. [45,46], who analyzed 2394 feet and similarly concluded that the calcaneo-stop procedure is associated with low complication rates and excellent functional outcomes. Compared with more invasive approaches such as medial displacement calcaneal osteotomy or subtalar fusion, the calcaneo-stop offers distinct advantages: it is reversible, preserves foot mobility, and can be performed as a day-surgery procedure with minimal morbidity [44,47].

However, its effectiveness appears to be most pronounced in flexible deformities in skeletally immature patients. In cases of rigid flatfoot, neuromuscular imbalance, or underlying coalitions, other techniques may be more appropriate [1,12].

The present findings should be interpreted considering the main limitations of the existing literature, including the predominance of retrospective designs, limited long-term follow-up, and marked variability in the outcome measures used. These factors hinder the ability to draw firm conclusions regarding the true effectiveness of arthroereisis. Nevertheless, this study adds value by providing a focused and detailed evaluation of the reported complications, thereby contributing to a more comprehensive understanding of the safety profile of the calcaneo-stop procedure.

### Limitations and future directions

This meta-analysis has some inherent limitations. Most included studies were retrospective, with heterogeneous follow-up durations and inconsistent reporting of complications. Only a minority used validated clinical scores or patient-reported outcome measures. Furthermore, definitions of complications – particularly minor ones such as incision discomfort – varied significantly between studies, introducing potential reporting bias. Interpretation of complication rates should consider the lack of standardized definitions and timing among the included studies. Postoperative pain and surgical site–related symptoms were variably defined, and some studies may have included early postoperative discomfort that would not necessarily qualify as a true complication. In addition, the assumption of zero events in studies with missing complication data, although applied conservatively and consistently, may have introduced bias, and potentially underestimated the true complication rate. Future research should prioritize prospective, multicenter studies with standardized complication definitions and include validated instruments for functional outcomes. Comparative trials between calcaneo-stop and other minimally invasive or open procedures may also help define more precise indications and optimize treatment algorithms.

### Clinical implications

From a clinical perspective, this study confirms that the calcaneo-stop technique represents a safe and effective solution for managing symptomatic pediatric FFF, particularly when performed in appropriately selected patients using optimal implant configurations. Careful consideration should be given to patient age, screw type, and diameter during surgical planning to minimize the risk of complications. Moreover, surgeons should anticipate transient postoperative symptoms and offer appropriate reassurance and management strategies to patients and families.

### Conclusion

The calcaneo-stop procedure is a safe and effective option for the surgical correction of symptomatic FFF in the pediatric population. This systematic review and meta-analysis, based on over 3000 treated feet, confirms a low overall complication rate, with most adverse events being mild and manageable.

Complication rates were influenced by factors such as screw type and diameter, as well as patient age, with older adolescents showing a higher risk of reintervention. These findings highlight the importance of appropriate implant selection and careful preoperative assessment.

Despite some limitations in the available literature, the evidence supports the calcaneo-stop technique as a valuable tool in pediatric orthopedic practice, offering reliable correction and high levels of patient and family satisfaction.

## Acknowledgements

### Conflicts of interest

There are no conflicts of interest.
